# Peri-implant Soft Tissue Phenotype Modification Using Free Gingival Graft and Apically Positioned Flap: A Case Series

**DOI:** 10.7759/cureus.102201

**Published:** 2026-01-24

**Authors:** Moustafa Saad, Joseph R Younes, Nadim Mokbel

**Affiliations:** 1 Periodontology, Private Practice, Tyre, LBN; 2 Periodontology, Saint Joseph University of Beirut, Beirut, LBN

**Keywords:** apically positioned flap, free gingival graft, keratinized mucosa width, peri-implant phenotype, periodontology

## Abstract

Background

The lack of peri-implant keratinized mucosa (<2 mm) is associated with plaque accumulation and pain during brushing, and consequently peri-implant disease. This case series aimed to evaluate the effectiveness of a free gingival graft in combination with an apically positioned flap to modify the peri-implant phenotype. The primary outcome was the change in keratinized mucosa width (KMW), and the secondary outcomes were pocket probing depth, plaque index, gingival index, pain upon brushing, and aesthetic appearance of the graft.

Methodology

A total of nine patients were treated with 16 implants placed in areas with a lack of KMW (<2 mm). After osseointegration and before the delivery of the final prosthesis, a free gingival graft in combination with an apically positioned flap was performed. All patients were followed up at 3, 6, and 12 months after surgery.

Results

The mean gain in KMW at 12 months was 2.51 mm. The change in KMW was statistically significant (p = 0.001). Plaque index and gingival index were reduced. Patients were satisfied with the aesthetic appearance and experienced less pain upon brushing.

Conclusions

Within the limitations of this case series, the use of a free gingival graft in combination with an apically positioned flap can modify the peri-implant phenotype by increasing the KMW and improving peri-implant health parameters.

## Introduction

The presence of a minimum amount of peri-implant keratinized mucosa (KM) around dental implants remains controversial [1,2]. While some researchers have shown that 2 mm of KM improves peri-implant health [3-5] and patients’ oral hygiene [6-8], as well as enhances the aesthetic appearance, others have reported that, in the presence of optimal plaque control, a smaller quantity of KM is required [2,9]. The two most commonly performed procedures to modify the peri-implant phenotype are the apically positioned flap (APF) technique, used alone or in conjunction with a free gingival graft (FGG) or a soft tissue substitute, and the bilaminar technique, which involves two layers of tissue, typically a connective tissue graft or soft tissue substitute, beneath a flap, either in a tunnel technique or with a coronally advanced flap [10-12]. The use of an autogenous free epithelialized mucosal graft is widely regarded as the gold standard for managing sites with absent or reduced keratinized mucosal width (KMW). This approach helps prevent the onset of the disease and the continuous deterioration of the architecture of the surrounding mucosa, while also reducing brushing discomfort around the implant sites. During implant exposure, APFs and FGGs are typically used to increase KMW. The most frequently used surgical approach is APF combined with an autogenous graft (APF-AG), typically harvested from the palatal mucosa [6,13,14]. This case series aimed to evaluate the effectiveness of APF combined with FGG in modifying the peri-implant phenotype.

## Materials and methods

Study population and sample size

A total of nine patients presenting with full or partial edentulism and exhibiting deficient keratinized tissue width (<2 mm) at the buccal aspect of 16 implants were included and treated in a private practice and followed up for one year. All patients were treated with an FGG at the second-stage surgery or after receiving the temporary restoration, but before the delivery of the final restoration. All surgical procedures were performed between January 2021 and April 2022 by a single operator (MS).

Ethics statement

All patients signed a consent form after an explanation of the surgical procedure and allowed the researchers to collect the personal data needed and perform the clinical evaluation. Ethical approval for this study was obtained from the Saint Joseph University of Beirut Ethics Committee (approval number: USJ-2024-111), in accordance with the Helsinki Declaration of 1975 (revised August 2018).

Inclusion and exclusion criteria

Patients were eligible for inclusion if they were older than 21 years; partially or fully edentulous, successfully treated with dental implants and ready for final prosthesis delivery; presenting with a keratinized tissue width <2 mm at the buccal aspect of the implant; no peri-implant disease; and exhibiting good oral hygiene, as verified by plaque index (PI) <1. Exclusion criteria included patients presenting with active periodontal disease, a smoking history of more than 10 cigarettes per day, those suffering from any systemic disease that interferes with oral surgery, non-compliant with the maintenance protocol, or those with malpositioned implants [15,16].

Surgical intervention

**Figure 1:**
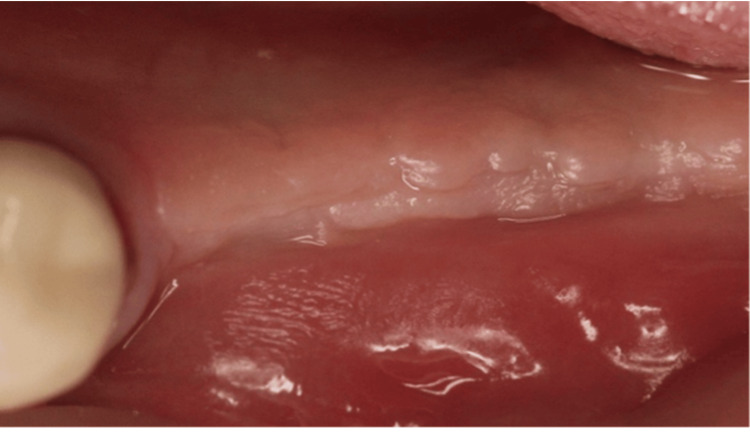
Clinical image showing a lack of keratinized mucosa buccally.

All surgical procedures were performed by a single, experienced clinician (MS). Following the administration of local anesthesia (4% articaine with 1:100,000 epinephrine; Septanest, Septodont, France), implants scheduled for second-stage surgery were exposed, and healing abutments of appropriate dimensions were installed. Figure 1 shows a case that was included in the study, with a lack of keratinized tissue, necessitating a second-stage surgery + FGG.

**Figure 2:**
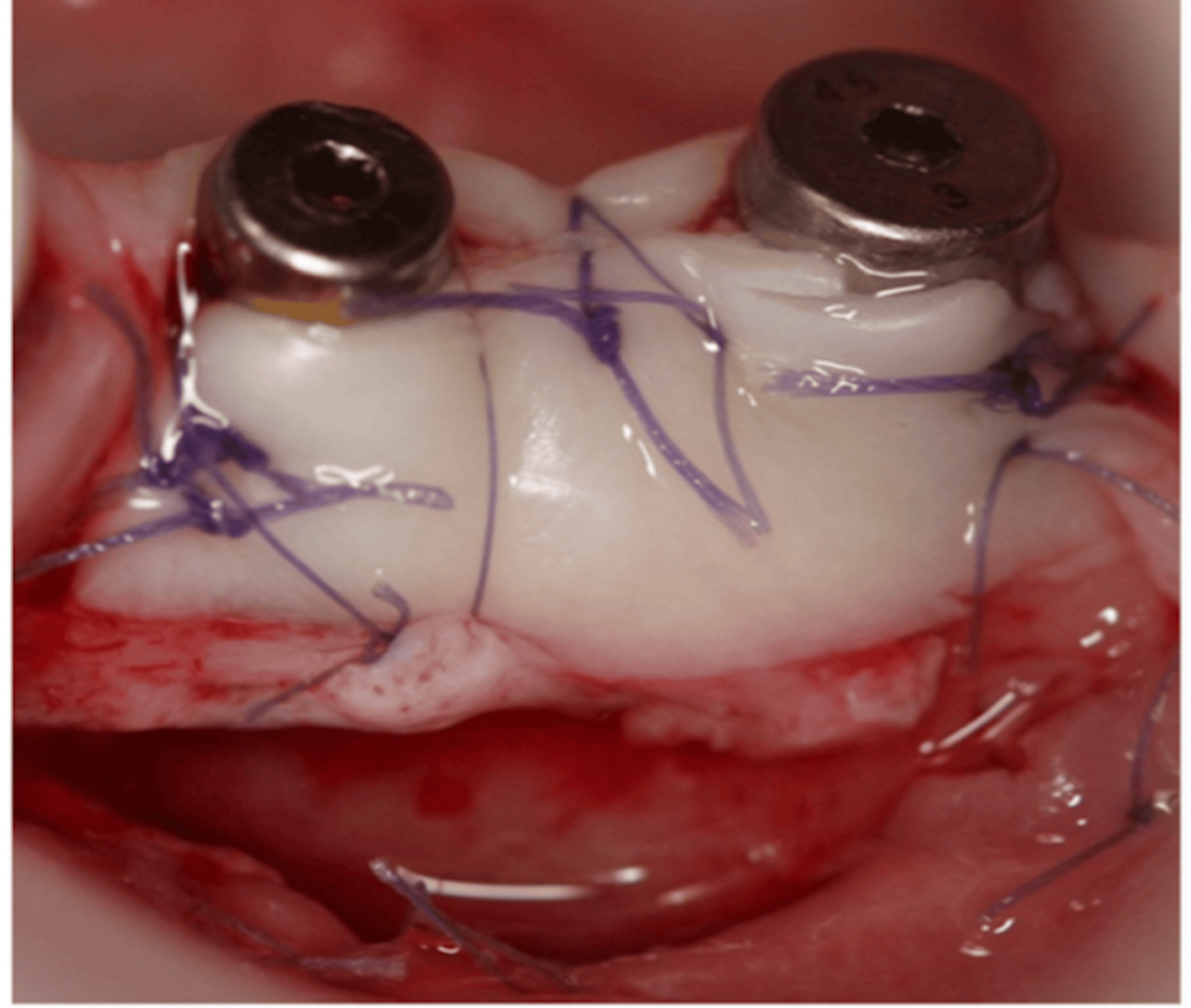
A free gingival graft secured in combination with connected healing abutments.

**Figure 3:**
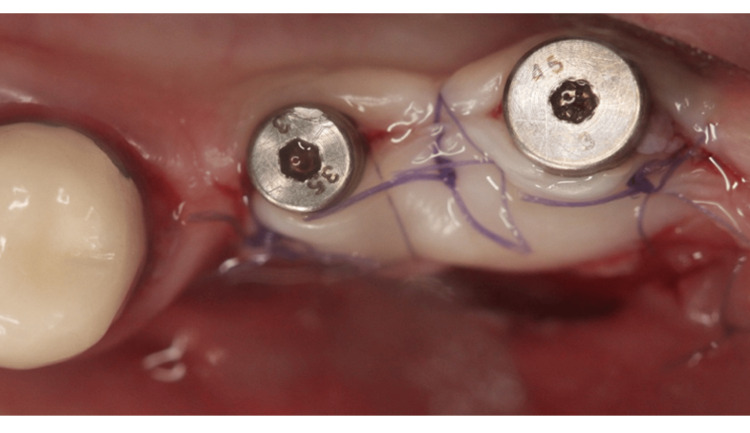
Occlusal view of the free gingival graft.

The recipient site was prepared using a No. 15C carbon steel blade (Henry Schein, NY, USA) by executing a horizontal split-thickness incision along the mucogingival junction (MGJ) on the buccal aspect of the implant site, complemented by two vertical releasing incisions positioned approximately 3 mm mesially and distally to the implant area. After detachment of the muscle fibers, the mucosal flap was apically repositioned and secured to the periosteum using 6-0 resorbable polyglycolic acid sutures (Omnia, Hu-Friedy, Chicago, IL, USA ). The required FGG dimensions were measured and harvested from the palatal mucosa using a 15C stainless steel blade (Henry Schein, NY, USA). A 2 mm dimension from the gingival margin of the neighboring teeth was measured before harvesting a 4 mm FGG of 1.2-1.5 mm thickness between the premolar and the molar area. Adipose and glandular tissues were removed from the graft. Its thickness was verified to be in harmony with the design. A collagen plug (Zimmer Collaplug, ZimVie Inc, USA) was secured with a horizontal mattress suture. A 5-0 resorbable polyglycolic acid surgical suture (Omnia, Hu-Friedy, Chicago, IL, USA) was placed to secure the blood clot. The FGG was then positioned and sutured to the periosteum at the recipient site with interrupted horizontal mattress sutures (5/0 and 6/0 resorbable polyglycolic acid sutures) (Figures 2, 3).

Postoperative instructions and infection control

**Figure 4:**
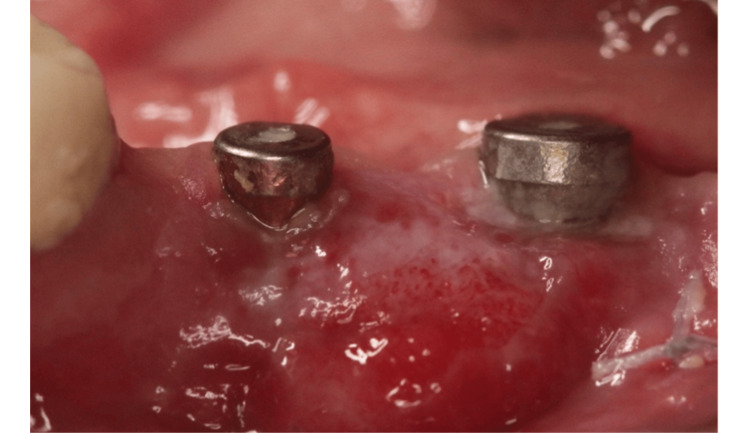
Image showing healing 14 days postoperatively.

An anti-inflammatory drug (ibuprofen) was prescribed immediately after surgery (1 tablet 600 mg three times per day) and subsequently, as needed. Patients were asked to rinse their mouths for one minute with a 0.12% chlorhexidine solution three to four times per day for 14 days. The sutures were removed between 10 days and 2 weeks postoperatively, and a maintenance program was established (Figure 4).

**Figure 5:**
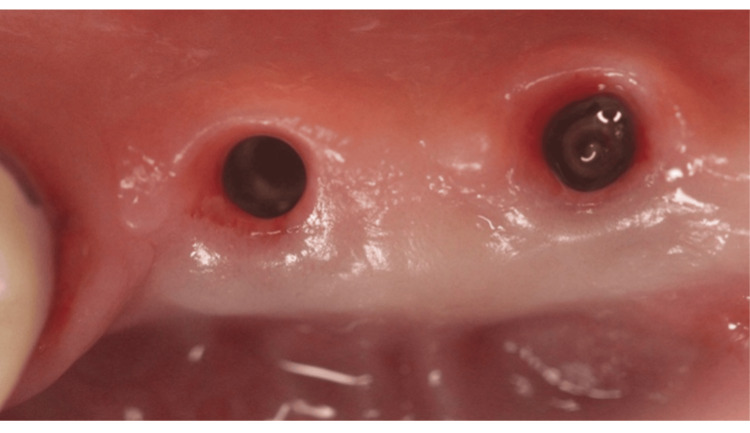
Three-month occlusal view following free gingival graft placement.

**Figure 6:**
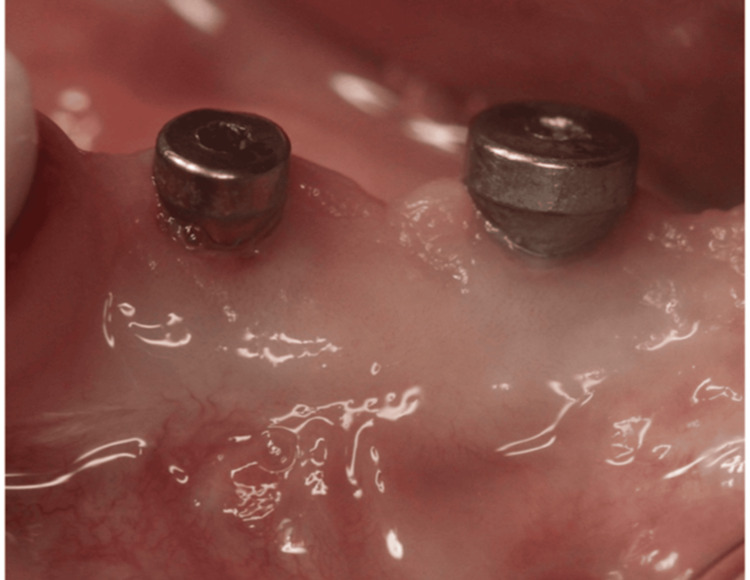
Three-month healing of a free gingival graft patient ready for prosthetic rehabilitation.

Postoperative follow-up visits were recommended at 12 weeks, 6 months, and 1 year after surgery to evaluate healing at both donor and recipient sites. Figure 5 and Figure 6 show the healing of the graft 12 weeks postoperatively.

Prosthetic phase

**Figure 7:**
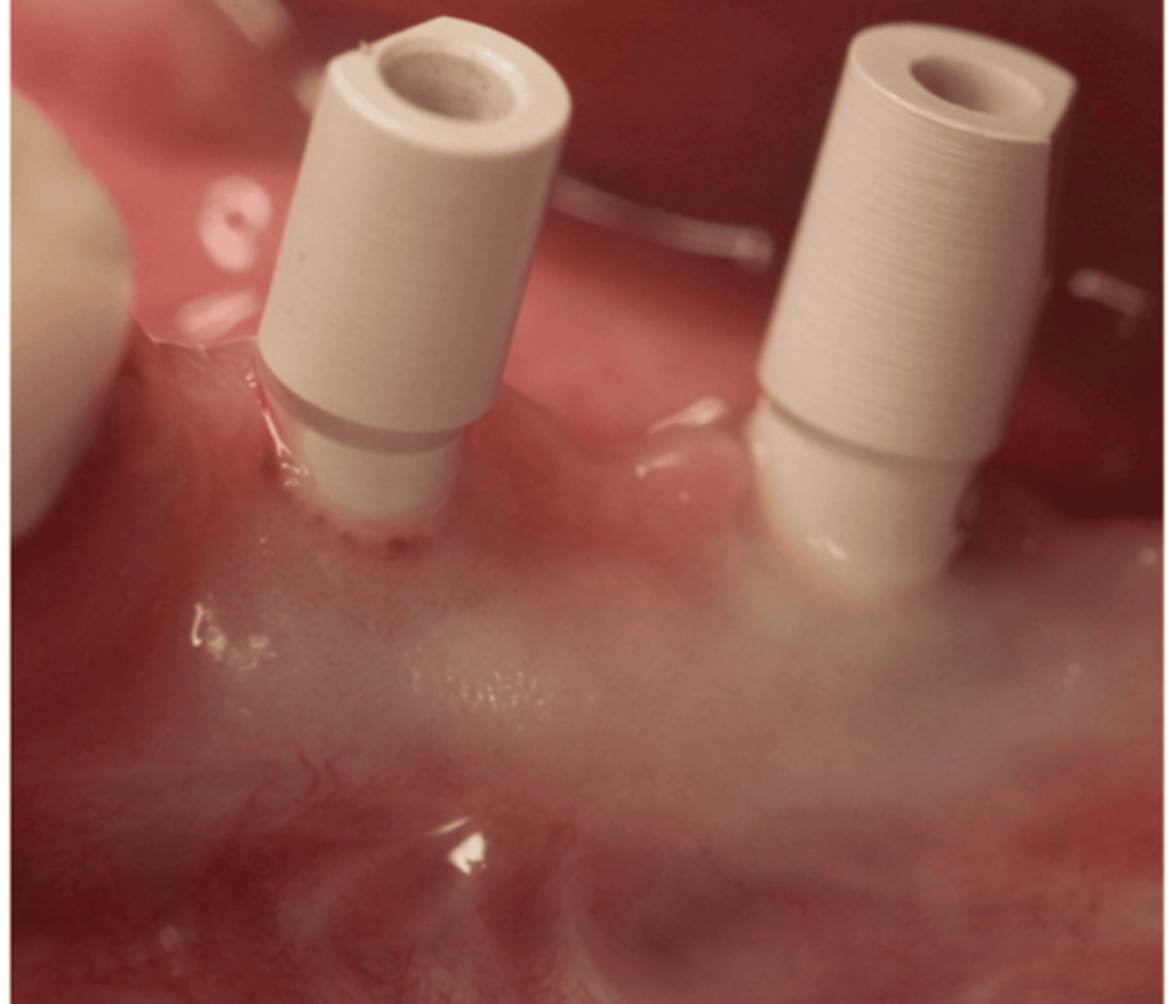
Scan body connected for digital impression.

Screw-retained metal-fused to ceramic crowns were fabricated after digital impressions (Figure 7).

**Figure 8:**
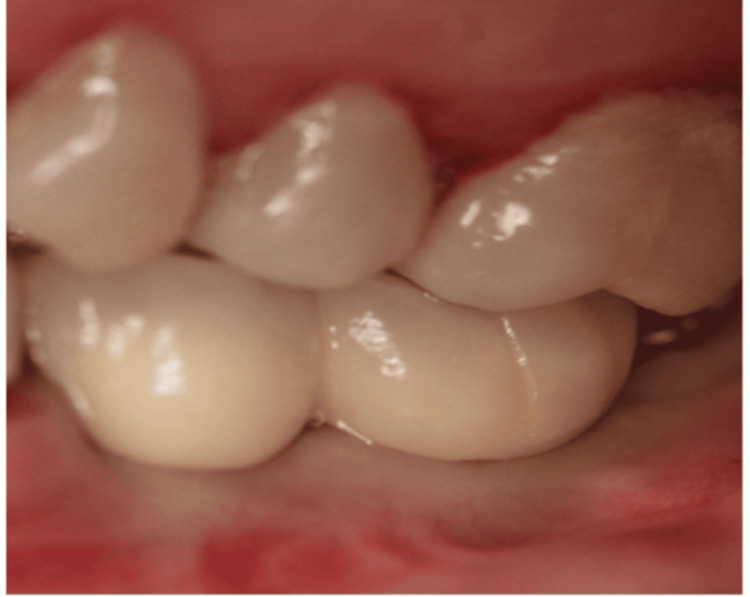
Crown delivery (centric occlusion).

Prosthetic crowns were placed three months later (Figure 8).

**Figure 9:**
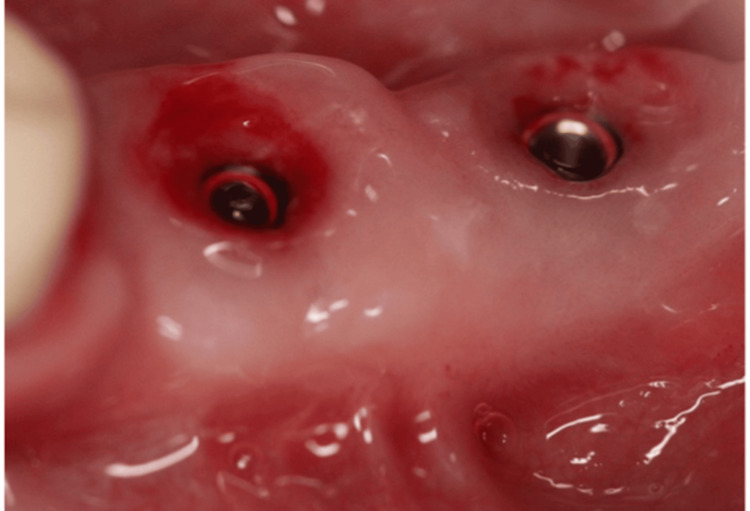
One-year frontal view showing 4 mm of keratinized mucosa buccally.

Clinical and radiographic evaluations were performed one year later. After the removal of the screw-retained crowns, an adequate band of KM was observed around the implants (Figure 9).

**Figure 10:**
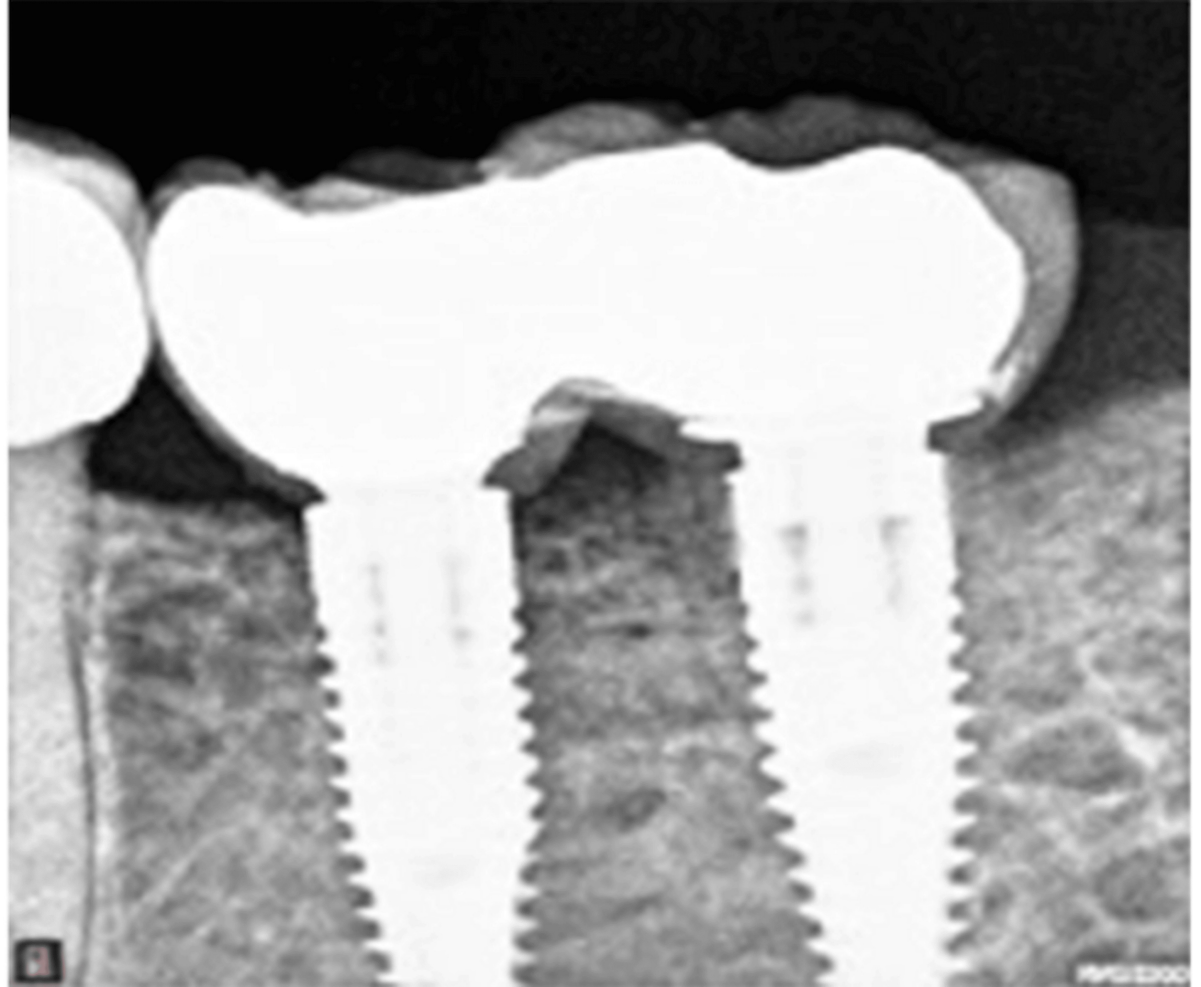
One year after loading.

On the peri-apical radiograph, stable marginal bone levels were observed (Figure 10).

Study measures/assessment of clinical peri-implant parameters

Before surgery, the KMW was measured using a periodontal probe (PCP 12PT, Hu-Friedy, Chicago, IL, USA) from the mucosal margin to the MGJ at the mid-buccal aspect of each implant site. The width of the keratinized tissue was further assessed using the rolling technique, measuring the distance between the free mucosal margin and the MGJ at the mid-buccal point. An experienced periodontist (NM) performed all KMW assessments at 3, 6, and 12 months postoperatively. The probing pocket depth (PPD) was recorded at the regular six sites of the implant after removal of the prosthesis, using a Michigan O color-coded probe (Hu-Friedy, Chicago, IL, USA). The gingival index (GI) (Silness and Löe) and PI (Silness and Löe) were also recorded at the four sites of the implant at the same evaluation intervals [17].

Outcome assessments

The primary outcome was the change in KMW (mm) at the buccal aspect of the treated implant sites at the 12-month follow-up. Secondary outcomes included PPD measurements, PI, GI, pain upon brushing, and the aesthetic appearance of the grafted area.

Statistical analysis

The statistical methodology was reviewed by an independent statistician. Data analysis was performed using SPSS Statistics version 20 (IBM Corp., Armonk, NY, USA). The implant served as the primary unit of analysis. The total number of patients and implants included in the study was reported, and means and standard deviations (SDs) were calculated for each of the four clinical variables, i.e., KMW, PPD, PI, and GI, at all four evaluation periods. A confidence level of 95% (α = 0.05) was adopted. Differences between time points were considered statistically significant at p-values <0.001. For PPD, one-way analysis of variance (ANOVA) with repeated measures was used to analyze the differences between the three time frames, where probing depth was measured multiple times at different time points (3 months, 6 months, and 12 months). We ensured that the data met both normality and sphericity (homogeneity of variances of the differences between all pairs of conditions).

Patient-reported outcomes

A numerical scale ranging from 0 to 4 was used to assess the intensity of pain upon brushing both preoperatively and postoperatively. A score of 0 indicated a complete lack of pain, and a score of 4 indicated the worst pain. To gain more insight into the patients’ satisfaction with the final aesthetic appearance, a questionnaire was administered, ranging from very dissatisfied to very satisfied.

## Results

Table 1 shows the distribution of patients according to age, sex, and implant site characteristics. The addressed patients (nine patients and 16 implants) had received dental implant therapy but had not yet proceeded to the final prosthetic stage.

**Table 1:** Patient’s distribution according to age, gender, implant site characteristics, and baseline keratinized mucosa width (KMW).

Patient number	Gender	Age in years	Implant site	Baseline KMW (mm)
1	Male	62	44	1
2	Female	65	43	1.1
			44	0.1
3	Female	59	44	0.2
			45	1.3
4	Female	66	16	0.1
			14	1.5
			32	0.3
5	Female	48	34	0.2
			35	1.1
6	Female	55	34	0
			36	0.2
7	Male	45	31	0
8	Female	28	31	1
9	Female	48	35	0
			36	0.2

The improvement of this surgical procedure on peri-implant health was evaluated by measuring the following parameters: KMW (primary outcome), PI, GI, and aesthetic appearance. Moreover, pain during brushing was assessed in the area where the procedure was performed. Table 2 shows the mean value of PI and GI at the four time frames. For PI and GI, the repeated measures ANOVA test indicated a non-significant difference at the four time frames (p = 0.4226). A reduction in the PI was observed from the baseline (0.95 mm) to 0.8 mm at 12 months. A similar observation was noted for GI. It was reduced from 1.1 mm (baseline) to 0.55 mm (12 months) (p < 0.001).

**Table 2:** Mean values of plaque index and gingival index at the four time frames.

	Baseline	3 months	6 months	12 months
Plaque index	0.95	0.9	0.9	0.8
Gingival index	1.1	0.7	0.8	0.55

**Figure 11:**
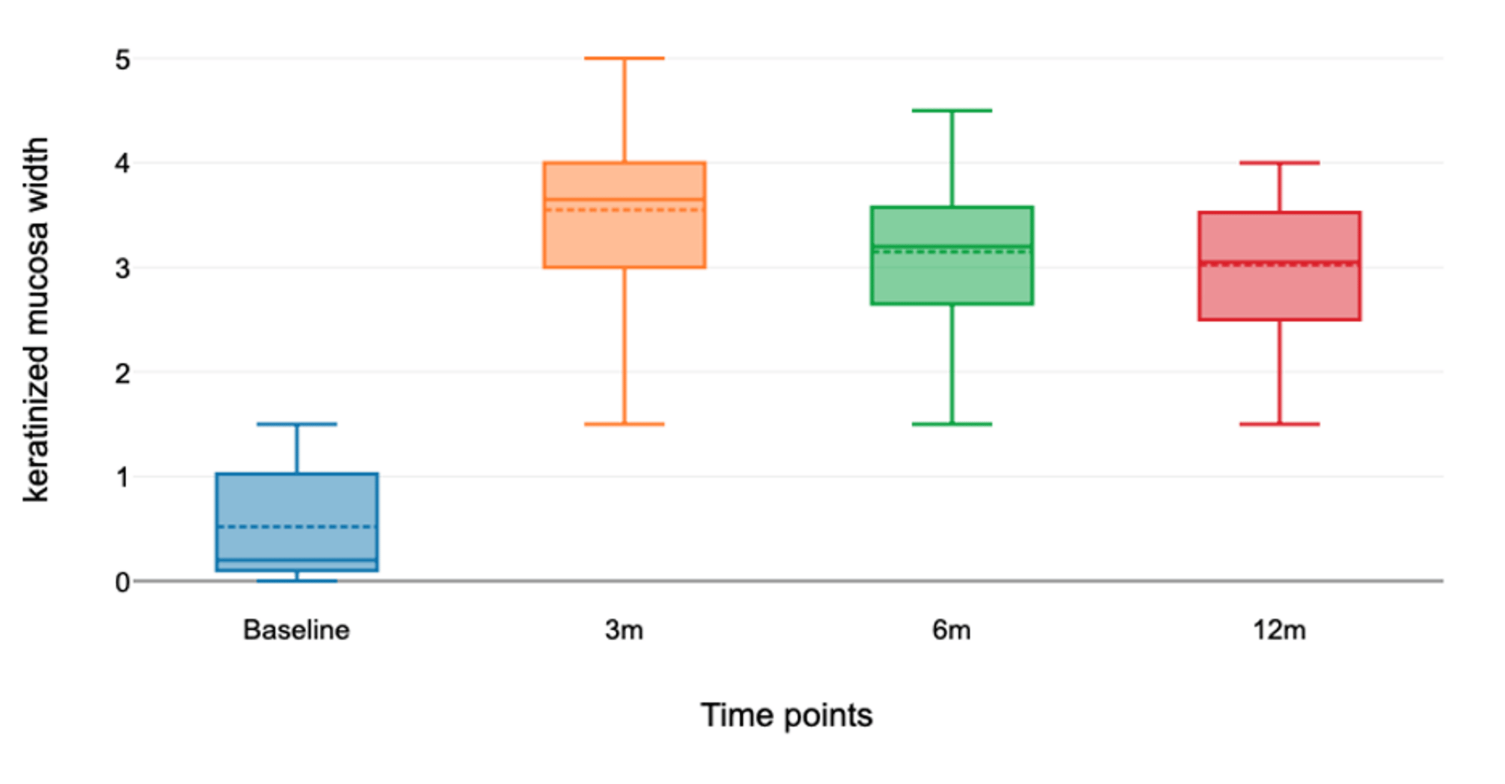
Boxplot comparing the medians at each time point. The figure shows that the median keratinized mucosa width after three months is higher than that at the baseline, as well as after 6 and 12 months of surgery.

Table 3 shows the mean KMW at baseline, 3 months, 6 months, and 1 year. The boxplot shown in Figure 11 compares the medians at each time point. The mean KMW was 0.52 mm at baseline, 3.55 mm at 3 months, 3.15 mm at 6 months, and 3.03 mm at 12 months. The Friedman test showed a significant difference between the mean KMW values at the four time points (p < 0.001). The gain in KMW from baseline to 12 months was 2.51 mm, with a p-value of 0.001, which was statistically significant (p < 0.00001).

**Table 3:** Mean keratinized mucosa width at baseline, 3 months, 6 months, and 12 months.

	Baseline	3 months	6 months	12 months
Mean	0.52	3.55	3.15	3.03
Median	0.2	3.65	3.2	3.05
Standard deviation	0.54	0.89	0.81	0.74
Minimum	0	1.5	1.5	1.5
Maximum	1.5	5	4.5	4

Table 4 shows the mean PPD at 3, 6, and 12 months. The mean scores at each time point were calculated and compared to identify significant differences. The mean PPD was 3.58, 3.56, and 3.57 mm at 3, 6, and 12 months, respectively. A mean reduction of 0.01 mm in PPD was observed, which was not statistically significant (p < 0.00001), as presented in Table 4.

**Table 4:** Mean pocket probing depth at 3 months, 6 months, and 12 months.

	3 months	6 months	12 months
Mean	3.58	3.56	3.57
Standard deviation	0.39	0.35	0.32
Minimum	3	3	3
Maximum	4.3	4	3.9
95% confidence interval of mean	3.38, 3.77	3.39, 3.73	3.41, 3.72

Regarding patients’ reported outcomes, pain intensity was significantly higher at the preoperative assessment (3.67 ± 0.47) than at the postoperative evaluation (0.55 ± 0.5) (p < 0.01), and the pain upon brushing was significantly lower postoperatively (Table 5).

**Table 5:** Assessment of post-surgical pain and postoperative pain upon brushing after three months. 0: no pain; 1: mild pain; 2: moderate pain, 3: very painful; 4: extreme pain.

	0	1	2	3	4
Post-surgical pain		Patients 3,9	Patients 1,4,5,6,7,8	Patient 2	
Pain upon brushing 3 months post-op	Patients 1,2	Patients 3,4,5,6,7,8,9			

Regarding aesthetic satisfaction, the questionnaire showed that 78% of the patients were very satisfied with the aesthetic outcome (Table 6).

**Table 6:** Patients’ satisfaction regarding the aesthetic results.

	Poor	Average	Good	Excellent
Are you satisfied with the aesthetic result?	-	Patient 7	Patient 3	Patients 1, 2, 4, 5, 6, 8, 9

## Discussion

The APF in combination with an FGG was used in this case series to evaluate the effectiveness of the technique in modifying the peri-implant phenotype. The mean gain in KMW from baseline was 2.51 mm with a p-value of 0.001, which was statistically significant. The mean width of KM was significantly increased in the nine treated patients at the three time points (3, 6, and 12 months) compared to the initial values preoperatively.

Many studies have assessed the effectiveness of FGG in increasing the amount of KMW around implants. Shah et al. (2021) reported a mean gain in the KMW of 3 ± 1.56 mm, 3.60 ± 0.79 mm, and 2.36 mm, respectively (six months post-surgery) [18]. Tavelli et al.’s (2021) findings are in accordance with ours [10]. They reported that the APF with FGG exhibited a significantly higher KMW gain compared to APF alone. In our study, a 4 mm apico-cervical dimension of FGG was used, while other authors, such as Shah et al. (2021) and Huang et al. (2021), did not mention the dimensions of the graft they harvested [18,19]. This small FGG may consequently reduce postoperative pain and improve patient comfort postoperatively.

When evaluating the PPD, a reduction in the measures was noted when compared to baseline, and they were maintained with no statistical differences throughout the three time frames (3, 6, and 12 months) in all nine patients. This can be explained by the adequate KMW that facilitates optimal oral hygiene measures by the patient and improves peri-implant health status. Huang et al. (2021) and Shah et al. (2021) also found stable PPD during the follow-up period with a mean probing depth of 2 ± 0.82 mm at six months [18,19]. The difference in PPD at six months without the study (3.56 mm) can be linked to the depth of implant placement and the initial patient phenotype of the peri-implant mucosa.

Regarding GI, there was a reduction in the measures when compared to the baseline, with no statistical differences in the three time frames (3, 6, and 12 months) among all nine patients. The increased amount of attached gingiva around the implants may have helped the patients in the cleaning process and improved their ability to remove plaque accumulation and decrease inflammation (less tissue attachment loss). Huang et al. (2021) and Thoma et al.’s (2017) findings are in accordance with ours [19-22]. In their studies, the groups treated with FGG showed a statistically significant reduction in GI index compared to the control group.

For patient-reported outcomes, two factors were assessed, i.e., pain intensity upon brushing and postoperative aesthetic appearance. Pain intensity was significantly higher at the preoperative assessment (3.67 ± 0.47) than at the postoperative evaluation (0.55 ± 0.5). This may be due to the increased thickness of the peri-implant mucosa after the FGG procedure, which may have increased resistance to the mechanical forces of the toothbrush. Regarding satisfaction with postoperative aesthetic appearance, the overall results showed that 78% of the patients in our study were very satisfied with the aesthetic outcome. Remarkably, older adults were much more satisfied than middle-aged adults. Indeed, the peri-implant mucosa is firmer, thicker, and covers the implant surface more than before; therefore, it is more esthetically pleasing to patients. Indeed, inter-patient differences in graft integration may reflect baseline phenotypic variability, as previously observed in soft tissue augmentation trials (Faour et al., 2022) [20].

In the present study, soft tissue management was performed before implant loading. To date, there is no clear consensus regarding the optimal timing or most effective technique for soft tissue augmentation around dental implants. However, evidence from multiple controlled clinical trials and meta-analyses indicates that FGG remains the most effective procedure for increasing KMW. Consistent with the latter findings, soft tissue augmentation in our study should be performed before loading. Similarly, Başeğmez et al. (2012) used FGG to manage implants exhibiting signs of mucositis. Furthermore, as suggested by Thoma et al. (2017), early intervention in soft tissue management may offer greater long-term benefits for maintaining peri-implant health [21,22]. However, some limitations are present, such as the small sample size and the heterogeneity of the included patients. A longer follow-up period is recommended to reduce the bias and generalize the conclusions.

## Conclusions

The FGG combined with an APF can modify the peri-implant phenotype by increasing KMW. Reducing the apico-coronal dimension of the FGG minimizes postoperative pain without compromising the gain of KM. Although PPD showed no significant change, and PI/GI reductions, while clinically favorable, were not statistically significant over time, other parameters of peri-implant health improved, consequently reducing the risk of peri-implant mucositis/implantitis. In our study, all patients were satisfied with the aesthetic appearance of the grafted area. More randomized clinical trials, including long-term follow-ups, are necessary to assess the stability of FGG and periodontal parameters over time.
